# Lactose Concentration in Low-Lactose and Lactose-Free Milk, Milk Products, and Products Containing Dairy Ingredients by High Sensitivity Enzymatic Method (K-LOLAC), Collaborative Study: Final Action 2020.08

**DOI:** 10.1093/jaoacint/qsac070

**Published:** 2022-06-11

**Authors:** Ruth Ivory, David Mangan, Barry V McCleary

**Affiliations:** Megazyme, Bray Business Park, Southern Cross Rd, Bray A98 YV29, Ireland; Megazyme, Bray Business Park, Southern Cross Rd, Bray A98 YV29, Ireland; Megazyme, Bray Business Park, Southern Cross Rd, Bray A98 YV29, Ireland

## Abstract

**Background:**

The AOAC Stakeholder Panel on Strategic Food Analytical Methods issued a call for methods in 2018 for the measurement of lactose in low-lactose and lactose-free products under *Standard Method Performance Requirement* (SMPR^®^) 2018.009. Megazyme’s Lactose Assay Kit (K-LOLAC) was reviewed and accepted as a First Action *Official Method*^SM^ in 2020 (**2020.08**).

**Objective:**

A collaborative study was conducted to evaluate the to evaluate the reproducibility of AOAC *Official Method*^SM^**2020.08** for the measurement of lactose concentration in low-lactose and lactose-free milk, milk products, and products containing dairy ingredients.

**Method:**

Samples are deproteinated and clarified by treatment with Carrez reagents, and then free glucose is removed using a glucose oxidase and catalase treatment system. Quantification of lactose is based on the hydrolytic activity of β-galactosidase, which hydrolyses lactose to glucose and galactose. Any remaining free D-glucose is first measured using a hexokinase (HK)/glucose 6-phosphate dehydrogenase (G-6PDH)/6-phosphogluconate dehydrogenase (6-PGDH) based assay procedure, and then β-galactosidase is added to hydrolyze the lactose in the same reaction vessel with concurrent measurement of the released D-glucose. The samples analyzed included a number of lactose-free and low-lactose milk samples, lactose-free infant formula, lactose-free milkshake, lactose-free adult nutritional drink, lactose-free cream, and lactose-free cheese.

**Results:**

All materials had repeatability relative standard deviations (RSD_r_) <7%. The reproducibility relative standard deviation (RSD_R_) varied from 3.8 to 14.9% with seven of the 10 test samples having an RSD_R_ of <10%.

**Conclusions:**

The Lactose Assay Kit (K-LOLAC) meets the requirements for reproducibility set out under SMPR 2018.009.

**Highlights:**

The Lactose Assay (K-LOLAC) is a robust, simple, and reproducible method for analysis of lactose in foodstuffs and beverages.

The measurement of lactose has been described using a range of methods including IR spectroscopy (AOAC *OfficialMethod*^SM^**972.16**) (1), polarimetry (AOAC *OfficialMethod*^SM^**896.01**) (2), and gravimetry (AOAC *OfficialMethod*^SM^**930.28**) (3) along with various types of chromatographic and enzymatic methods (4–6). Of these, only chromatographic and enzymatic methods can exhibit the selectivity and sensitivity required for the accurate measurement of lactose in low-lactose or lactose-free products (7–9).

Traditional enzymatic methods have relied on the β-galactosidase-mediated hydrolysis of lactose and the subsequent measurement of the glucose or galactose released as a result (10, 11). These methods are rapid, easy to perform, and highly cost-effective. They are well suited to the measurement of lactose in traditional dairy products in which lactose contents of ∼5 g/100 mL are usually found. However, due to the presence of galactosyl-glucosyl oligosaccharides in lactose-free samples (some of which have the potential to interfere by inflating glucose or galactose released upon hydrolysis with β-galactosidase), the application of traditional enzymatic tests to the measurement of lactose in lactose-free products typically results in inaccurate quantitation, with overestimation of the residual lactose present being the most prevalent outcome (12). This necessitates the utilization of a β-galactosidase enzyme that is selective for lactose in the presence of these potentially interfering sugars.

Megazyme’s Lactose Assay (K-LOLAC) is an enzymatic method used for the rapid measurement of lactose in foodstuffs and beverages. This procedure is simple, accurate, and sensitive, and it is suitable for the determination of lactose in low-lactose or lactose-free products, including infant formula and adult nutritional drinks, conventional dairy samples, and a variety of food samples. Quantification is based on the hydrolytic activity of β-galactosidase, which hydrolyses lactose to glucose and galactose. The β-galactosidase employed is selective for lactose, and the determined lactose values are similar to those obtained by high-performance anion exchange chromatography with pulsed amperometric detection (HPAEC-PAD) (13).

A single laboratory validation was previously reported (7) with analysis performed on 36 different commercial food and beverage products and a set of 10 certified reference materials. Parameters examined during the validation included working range and linear range, selectivity, limit of detection (LOD), limit of quantification (LOQ), trueness (bias), precision (repeatability and intermediate precision), robustness, and stability. The method was reviewed against *Standard Method Performance Requirement* (SMPR^®^) 2018.009 (14) and was accepted for First Action status with the method number **2020.08** (15). A multi-laboratory collaborative study involving 13 laboratories was conducted and is discussed herein.

##  

### Collaborative Study

Four (coded) homogeneous samples were provided as eight blind duplicates to collaborating laboratories as a familiarization sample set. This sample set included one lactose-free milk, one low-lactose milk, one lactose-free cream, and one lactose-free cheese sample. Subsequently, 10 (coded) homogeneous samples were provided as 20 blind duplicates to a total of 13 collaborating laboratories as the full sample set. This sample set included four lactose-free milks, two low-lactose milks, one lactose-free milkshake, one lactose-free adult nutritional drink, and two lactose-free infant formula samples. To facilitate the selection of the appropriate sample treatment and sample volume, collaborators were advised if the sample was “lactose-free” or “low-lactose.” Collaborators were requested to perform single extractions on each material by the enclosed method, but duplicate assays on aliquots of test material at the measurement step were requested. Results were evaluated according to AOAC Guidelines and using an AOAC calculations spreadsheet. Outlier results identified by the Cochran test for extremes of repeatability and the Grubbs test for extremes of reproducibility were omitted from further calculations. Also determined were within (s_r_) and between (s_R_) laboratory standard deviations, repeatability (r) and reproducibility (R) as 2.8 × s_r_ and 2.8 × s_R_, respectively, and relative standard deviations (RSD_r_) and (RSD_R_) from s_r_ and s_R_ as percentages of mean values.

## AOAC *Official Method*^SM^ 2020.08

**Lactose Concentration in Low-Lactose and Lactose-Free Milk, Milk Products, and Products Containing Dairy**

**Ingredients High-Sensitivity Enzymatic Method**

**Lactose Assay Kit (K-LOLAC) First Action 2020**

**Final Action 2021**

[Applicable for determination of lactose in low-lactose or lactose-free products, including infant formula and adult nutritional drinks, conventional dairy samples, and a variety of food samples in the range of 0.89–250 mg/100 mL for liquid samples and 1.47–430 mg/100 g for solid samples. Some overestimation of lactose concentration may occur in samples containing 1,3-β-D-galactosyl glucose and 1,3-β-D-galactosyl lactose.]

*Caution:* Adhere to general safety measures that apply to all chemical substances. For more information regarding safe usage and handling of reagents and components, refer to associated safety data sheet available from the manufacturer’s website (www.megazyme.com). The test should not be carried out by anyone other than trained and experienced laboratory analysts. Include lactose control with analysis if there is any doubt as to the performance of reagents or analyst.

### A. Principle

The enzymatic method relies on the β-galactosidase mediated hydrolysis of lactose and the subsequent measurement of the glucose released. The method describes the selective hydrolysis of lactose by MZ104 β-galactosidase, with further selectivity achieved by use of a linear extrapolation calculation to remove the contribution from interfering sugars commonly found in lactose-free samples, such as β-1,6-lactose (allolactose), that are slowly hydrolyzed during the incubation.

Free D-glucose is efficiently removed from the sample by conversion to D-gluconic acid using a glucose oxidase/catalase pretreatment system. Prior to lactose hydrolysis, any remaining free D-glucose is phosphorylated by the enzyme hexokinase (HK) in the presence of adenosine-5′-triphosphate (ATP) to glucose-6-phosphate (G-6-P) with the simultaneous formation of adenosine-5′-diphosphate (ADP). G-6-P is oxidized by the enzyme glucose-6-phosphate dehydrogenase (G6P-DH) in the presence of nicotinamide-adenine dinucleotide phosphate (NADP^+^) to gluconate-6-phosphate (gluconate-6-P) with the formation of reduced nicotinamide-adenine dinucleotide phosphate (NADPH). Gluconate-6-P is immediately converted to D-ribulose-5-phosphate (R-5-P), carbon dioxide (CO_2_), and a further molecule of NADPH by the enzyme 6-phosphogluconate dehydrogenase (6-PGDH). Lactose is then hydrolyzed to D-galactose and D-glucose by β-galactosidase, and the D-glucose released enters the series of reactions catalyzed by HK, G6P-DH, and 6-PGDH. The amount of NADPH formed is stoichiometric to twice the amount of lactose as two molecules of NADPH are produced for each D-glucose molecule originating from the lactose in the sample.

### B. Equipment

- *Volumetric flasks and glass beakers.—*50 and 100 mL.
- *Disposable plastic microfuge tubes.—*2 mL.
- *Disposable polypropylene tubes.—*13 mL.
- *Disposable plastic cuvettes.—*1 cm light path, 1.5 mL.
- *Micro-pipettors.—*e.g., Gilson Pipetman^®^ P20 and P100.
- *Analytical balance.*
- *Magnetic stir plate with heating capability.—*Required maximum temperature 70°C.
- *Boiling water bath.—*Required temperature 100°C.
- *Microfuge.—*Required speed 10 000 × *g*.
- *UV-VIS spectrophotometer*.—Required wavelength 340 nm.
- *Vortex mixer.*
- *Filter papers.—*e.g., Whatman No. 1, 9 cm.

### C. Chemicals and Reagents

Reagents are supplied in the Megazyme Lactose Assay Kit (K-LOLAC).

- *Solution 1.—*Bottle 1 containing buffer (pH 8.0), MgCl_2_, plus sodium azide (0.02%, w/v) as a preservative. Use the contents of Bottle 1 as supplied. Stable for >3 years at 4°C.
- *Solution 2.—*Bottle 2 containing glucose oxidase and catalase, lyophilised powder. Stable for >3 years below –10°C. For use, dissolve the contents of Bottle 2 in 14 mL of distilled water. To avoid repetitive freeze/thaw cycles, divide into appropriately sized aliquots and store in polypropylene tubes below –10°C. Stable for >3 years below –10°C.
- *Solution 3.—*Bottle 3 containing buffer (pH 7.6), MgCl_2_, and KCl plus sodium azide (0.02%, w/v) as a preservative. Use contents of Bottle 3 as supplied. Stable for >3 years at 4°C.
- *Solution 4.—*Bottle 4 containing NADP^+^, ATP, PVP, and mannitol plus sodium azide as a preservative. Stable for >3 years below –10°C. For use, dissolve the contents of Bottle 4 in 4 mL of distilled water. To avoid repetitive freeze/thaw cycles, divide into appropriately sized aliquots and store in polypropylene tubes. Stable for >3 years below –10°C.
- *Suspension 5.—*Bottle 5 containing hexokinase, glucose 6-phosphate dehydrogenase, and 6-phosphogluconate dehydrogenase plus sodium azide (0.02%, w/v) as a preservative. Use the contents of Bottle 5 as supplied. Before opening for first time, shake the bottle to remove any enzyme that may have settled on the stopper. Subsequently store the bottle in an upright position. Swirl the bottle to mix contents before use. Stable for >3 years at 4°C.
- *Suspension 6.—*Bottle 6 containing MZ104 β-galactosidase suspension plus sodium azide (0.02%, w/v) as a preservative. Use the contents of Bottle 6 as supplied. Before opening for the first time, shake the bottle to remove any enzyme that may have settled on the stopper. Subsequently store bottle in an upright position. Swirl bottle to mix contents before use. Stable for >3 years at 4°C.
- *Solution 7.—*Bottle 7 containing lactose standard solution (25 mg/100 mL) plus sodium azide (0.02%, w/v) as a preservative. Use contents of Bottle 7 as supplied. Stable for >3 years at 4°C.
  Additional reagents (not supplied in Lactose Assay Kit):
- *Concentrated Carrez I solution.—*200 mL. Dissolve 30 g potassium hexacyanoferrate (II) trihydrate (K_4_[Fe(CN)_6_]·3H_2_O) in 200 mL distilled water. Stable for >3 years at room temperature.
- *Concentrated Carrez II solution.—*200 mL. Dissolve 60 g of zinc sulphate heptahydrate (ZnSO_4_·7H_2_O) in 200 mL distilled water. Stable for >3 years at room temperature.
- *Hydrogen peroxide (H_2_O_2_).—*Approximately 30% (w/w). Use as purchased. Refer to manufacturer guidelines for safety and stability information.

### D. Sample Preparation

- *Sample reconstitution procedure for infant formula, whey protein, adult nutritional drinks, and other samples intended to be consumed in beverage form.—*Solid samples that are intended to be consumed in liquid form should be prepared in liquid form before testing in the (b) *Sample Extraction/Clarification Procedure: Liquid Samples* procedure. A sample preparation example is outlined below:
  1. Accurately weigh 7.5 g powdered sample into a 50 mL glass beaker. Record the weight.
  2. Add a stir bar and approximately 30 mL of distilled water at ∼40°C.
  3. Mix on a magnetic stir plate for approximately 15 min.
  4. Quantitatively transfer to a 50 mL volumetric flask.
  5. Dilute to volume (50 mL) with distilled water.
  6. Take 0.5 mL of liquid sample for the (b) *Sample Extraction/Clarification Procedure for Liquid Samples* procedure.
- *Sample extraction/clarification procedure for liquid samples*.—
  1. Ensure that the milk sample has been mixed thoroughly before sampling.
  2. Pipet the following reagents into a 2 mL disposable microfuge tube: 0.9 mL of distilled water (at room temperature, ∼20–25°C); 0.5 mL milk sample; 0.05 mL Carrez II solution; 0.05 mL Carrez I solution.
  3. Cap tube and mix by vortex.
  4. Centrifuge at 10 000 × *g* for 10 min.
  5. Take 1.0 mL clear filtrate for (e) *Glucose oxidase/catalase pretreatment procedure*.
- *Sample extraction procedure for solid samples.—*
  1. Accurately weigh approximately 10 g solid sample into 50 mL glass beaker.
  2. Add stir bar and approximately 30 mL distilled water.
  3. Mix on magnetic stir plate and heat until temperature reaches 50 °C. Continue stirring at temperature for approximately 15 min or until sample has solubilized or homogenized.
  4. Quantitatively transfer to 50 mL volumetric flask.
  5. Add 0.5 mL Carrez II solution and mix.
  6. Add 0.5 mL Carrez I solution and mix.
  7. Make to volume (50 mL) with distilled water.
  8. Filter an aliquot, discarding the first few mL of filtrate (∼5 mL).
  9. Take 1.0 mL clear filtrate for the (e) *Glucose Oxidase/Catalase Pretreatment Procedure*.
- *Solubilization and extraction procedure for cheese*.—The melting point of cheese can vary based on parameters such as moisture content and age. Perform solubility assessment by visual inspection. Although the solution may not be free of turbidity after solubilization, there should be no lumps present or noticeable lack of homogeneity. A sample preparation example is outlined below:
  1. Using a standard kitchen hand-grater, grate cheese through a fine sieve.
  2. Accurately weigh approximately 10 g of grated sample into a 50 mL glass beaker.
  3. Add stir bar and approximately 30 mL distilled water.
  4. Mix on magnetic stir plate and heat until temperature reaches 50°C. Continue stirring at temperature for approximately 15 min and assess the level of solubilization.
  5. If cheese has not solubilized at 50°C, slowly increase temperature by 5°C and further assess the level of solubilization.
  6. Continue to increase the temperature in increments of 5°C up to a temperature of 70°C if solubilization or homogenization has not occurred at lower temperatures.
  7. Quantitatively transfer to a 50 mL volumetric flask.
  8. Add 0.5 mL Carrez II solution and mix.
  9. Add 0.5 mL Carrez I solution and mix.
  10. Dilute to volume (50 mL) with distilled water.
  11. Filter an aliquot, discarding the first few mL of filtrate (∼5 mL).
  12. Take 1.0 mL clear filtrate for the (e) *Glucose Oxidase/Catalase Pretreatment Procedure*.
- *Glucose oxidase/catalase pretreatment procedure for all samples*.—
  1. Pipet the following reagents to a 13 mL polypropylene tube: 0.4 mL distilled water (at room temperature, ∼20–25°C); 1.0 mL clear supernatant; 0.1 mL Solution 1; 0.2 mL Solution 2; 0.1 mL H_2_O_2_ (∼30%, w/w).
  2. Cap the tube and mix by vortex.
  3. Incubate at room temperature (20–25°C) for 15 min.
  4. Slowly loosen the cap to release pressure and then retighten.
  5. Incubate in a boiling water bath (100°C) for 5 min.
  6. Remove from the water bath and allow to cool for approximately 5 min.
  7. Transfer approximately 1.0 mL of solution to a 2 mL microfuge tube and centrifuge at 10 000 × *g* for 10 min.
  8. Carefully pipet the required volume (0.1–0.4 mL) for use in the (f) *Enzymatic Determination Reaction* procedure. Note that the volume pipetted depends on the sample type. For lactose-free samples, the maximum volume of 0.4 mL should be transferred. For low-lactose samples, a volume of 0.1 mL should be transferred.
- *Enzymatic determination reaction for all samples.—*Note that for sample cuvettes, the amount of distilled water added should be adjusted depending on the sample volume required (i.e., 0.9 mL distilled water where 0.1 mL of sample is used and 0.6 mL distilled water where 0.4 mL of sample is used).
  1. Set the spectrophotometer to read absorbance at 340 nm.
  2. Blank spectrophotometer against air or water.
  3. Prepare a blank cuvette by addition of the following reagents to a 1.5 mL UV cuvette: 1.0 mL of distilled water; 0.1 mL of Solution 3; 0.05 mL of Solution 4.
  4. Cap the cuvette using a cuvette cap or parafilm and mix by gentle inversion.
  5. Prepare sample cuvettes (in duplicate) by addition of the following reagents to a 1.5 mL UV cuvette: 0.9 mL or 0.6 mL distilled water; 0.1 mL or 0.4 mL sample solution [from the (e) *Glucose Oxidase/Catalase Pretreatment* procedure]; 0.1 mL of Solution 3; 0.05 mL of Solution 4.
  6. Recap the cuvette using a matching cuvette cap or parafilm and mix by gentle inversion.
  7. If required, prepare a lactose standard cuvette by addition of the following reagents to a 1.5 mL UV cuvette: 0.9 mL distilled water; 0.1 mL Solution 7; 0.1 mL Solution 3; 0.05 mL Solution 4.
*Note*: The lactose standard solution (Solution 7) is assayed only where there is some doubt about the accuracy of the test (e.g., issues with analyst, equipment, or reagents).
  8. Recap the cuvette using a matching cuvette cap or parafilm and mix by gentle inversion.
  9. After 3 min at 25°C, read absorbances of blank and then samples (A_1_).
  10. Remove cuvette caps or parafilm, taking care to avoid spillage of liquid.
  11. Start reaction by addition of 0.02 mL Suspension 5. Recap cuvettes with matching cap or parafilm after addition and mix by gentle inversion.
  12. After 10 min at 25°C, read absorbances of blank and samples (A_2_).
  13. Remove cuvette caps or parafilm, taking care to avoid spillage of liquid.
  14. Start the next reaction by addition of 0.02 mL of Suspension 6. Recap cuvettes with matching cap or parafilm after addition and mix by gentle inversion.
  15. After 15 min at 25°C, read the absorbances of blank and samples (A_3_).
  16. After a further 5 min (20 min from addition of Suspension 6) at 25°C, read absorbances of blank and samples (A_4_).
  17. After a further 5 min (25 min from addition of Suspension 6) at 25°C, read absorbances of blank and samples (A_5_).

### E. Calculations

Determine the value for A_3_ “creep corrected” (A_3cc_) using the MegaCalc software available on the manufacturer’s website, or equation of line calculation below. A_3cc_ can be calculated as follows:

First, calculate slope of line (m)

$$m=\left(y_{2}–y_{1}\right)/\left(x_{2}–x_{1}\right)$$

where *y*_2_ = absorbance value A5 (measured after 25 min); *y*_1_ = absorbance value A3 (measured after 15 min); *x*_2_ = time of measurement for A5 reading (25); and *x*_1_ = time of measurement for A3 reading (15).

Then calculate the *y*-intercept *A*_3cc_

$$A_{3\text{cc}}=y–mx$$

where *y* = absorbance at *A*_3_; *x* = time of measurement *A*_3_ (15).

Use the value for *A*_3cc_ in the calculation of Δ*A*_lactose_ below.

Determine the absorbance difference caused by hydrolysis of lactose (*A*_3cc_ — *A*_2_) for both blank and sample. Subtract the absorbance difference of blank from the absorbance difference of sample, thereby obtaining Δ*A*_lactose_. The value of Δ*A*_lactose_ should as a rule be in the range of 0.02–1.2 absorbance units to achieve sufficiently accurate results.

Concentration of lactose can be calculated in mg/100 mL (w/v) as follows:

$$\begin{array}{c} C_{\text{lactose}}=\left[\left(V\times MW\right)/\left(\varepsilon \times d\times v\times 2\right)\right]\\ \times \Delta A_{\text{lactose}}\times 1\mathrm{00}\times F \end{array}$$

where *V* = enzymatic determination reaction final volume, mL, 1.19; MW = molecular weight of lactose, g/mol, 342.3; ε = extinction coefficient of NADPH at 340 nm (l × mol^−1^ × cm^−1^), 6300; d = light path, cm, 1; v = sample volume, mL, 0.1 or 0.4; 2 = 2 moles NADPH produced for each mole of D-glucose or lactose; 100 = conversion to mg/100 mL; F = dilution factor.

For reconstituted solid samples (a) and liquid samples (b), F is calculated as follows:

$$\begin{array}{c} F=\left(V_{1}/v_{1}\right)\times \left(V_{2}/v_{2}\right)\\ F=\left(1.5/0.5\right)\times \left(1.8/1\mathrm{.0}\right)=5.4 \end{array}$$

where *V*_1_ = final volume in liquid extraction procedure, mL; *v*_1_ = sample volume in liquid extraction procedure, mL; *V*_2_ = final volume in glucose oxidase/catalase treatment, mL; *v*_2_ = sample volume in glucose oxidase/catalase treatment, mL.

For solid sample extraction (c) and cheese sample extraction (d), F is calculated as follows:

$$\begin{array}{c} F=V_{2}/v_{2}\\ F=1.8/1\mathrm{.0}=1.8 \end{array}$$

where *V*_2_ = final volume in glucose oxidase/catalase treatment, mL; and *v*_2_ = sample volume in glucose oxidase/catalase treatment, mL.

For reconstituted and solid samples, to express as mg/100g (w/w) as follows:

$$\text{mg}/1\mathrm{00}\;g=\left[c_{\text{lactose}}\text{/extract concentration}\right]$$

where c_lactose_ is expressed in mg/100 mL as calculated above;

$$\begin{array}{cc} \text{extract concentration} & =7.5\;g/50\;\text{mL}=0.15\;g/\text{mL}\;\left(\text{reconstituted samples}\right)\\ & =10\;g/50\;\text{mL}=0.2\;g/\text{mL}\;\left(\text{solid samples}\right) \end{array}$$

(Calculations can be greatly simplified using the Excel-based calculator available on the Megazyme website where the product is listed.)

## Results

### Sample Selection

Sample selection for this study proved to be a challenge. Initially, stability tests on all samples were carried out prior to selection for the study, and those samples exhibiting poor stability (over a period of 3–4 weeks) were excluded from the sample set. As would be expected for dairy ingredient–based samples, medium- to long-term stability proved to be an issue across many of the samples evaluated.

In addition to sample stability issues, difficulties arose when screening samples for lactose at the lactose-free level, with many samples reporting as “below LOQ” when analyzed. This was particularly evident in the case of lactose-free cheese samples, where multiple samples were screened but no sample was identified that contained lactose at a level above the LOQ of the method. Inclusion of a cheese sample in the multi-laboratory evaluation was desirable to show that despite recognized difficulties in sample handling, the observed assay reproducibility nonetheless remained at an acceptable level.

The pre-trial familiarization analysis provided important data and insights that helped to dictate the course of the study. Samples selected for pre-trial analysis were lactose-free milk, low-lactose milk, lactose-free cream, and lactose-free cheese. The milk samples generally returned good reproducibility. The lactose-free cream returned good single-laboratory repeatability data; however, this sample caused issues when analyzed by collaborators. Multiple collaborators reported issues with a lack of homogeneity of the sample apparent on arrival, leading to the conclusion that the stability of this sample type was not sufficient when considering delays that may occur during international shipping. A lactose-free cheese sample was included in the pre-trial sample set in order to broaden the sample set and evaluate the reliability of the method below the LOQ. All collaborators returned values for this material below the LOQ, and although this information is valuable, the decision was made not to carry this sample through to the full sample set as limited further information could be gained by its inclusion. The pre-trial data set is not shown.

One collaborator contacted the study director to request that a lactose-free product (a commercially available milk product) that was produced by a “classical” lactase be included in the main sample set. The collaborator requested that the sample contain a lactose concentration below the commonly used lactose-free threshold (<10 mg/100 g). As lactose-free products produced using so-called classical lactases (i.e., lactase from *Kluyveromyces lactis*) represent a significant part of the lactose-free market, this request was facilitated by the study director. A commercially available lactose-free milk sample (containing <10 mg/100 g lactose), manufactured by a brand specified by the collaborator, was included in the sample set (sample 6VVU/273B).

AOAC multi-laboratory study guidelines outlined in Appendix D (16) recommend that samples are chosen to cover the concentration range of interest. For the full sample set, aforementioned difficulties in finding samples with a suitable intrinsic lactose concentration led to the decision to prepare a panel of samples containing the required levels of lactose. As milk was deemed to be the most commercially relevant sample type, an attempt was made to include this particular sample type with a range of lactose concentrations. Appendix D states that “Spiked materials consisting of normal or blank materials to which a known amount of analyte has been added may be used.” Samples B24Z/QVHG, DL4A/EAN7, and C886/BBJ3 were prepared by addition of lactose to various lactose-free commercial milk products. A number of unspiked milk products were also included in the sample set (6VVU/273B, JAV2/6C6R, 575E/Q2QA). In addition to the milk samples, two lactose-free infant formula powder samples (YJ2E/WYB8 and 2NUB/4JDP), one lactose-free milkshake (T3RC/3VXP), and a lactose-free adult nutritional drink powder (ZTB2/N2Z6) were included in the sample set.

### Collaborator Data

Collaborators’ data were evaluated statistically according to AOAC protocols using AOAC-supplied software, and results are summarized in Tables 1–3. Data from Laboratories 3 and 7 were removed from the statistical evaluation of the method as both laboratories reported issues with the analysis. In the case of Laboratory 3, the A_2_ absorbance values for a number of samples were unacceptably high, suggesting that the glucose oxidase and catalase pretreatment was not performed correctly. Laboratory 7 reported issues with the clarification of the sample and suggested that an incorrect volume of Carrez reagents may have been used for some of the samples analyzed. Both laboratories offered to repeat the testing; however, this was not deemed to be necessary after results processing showed that all other laboratories had returned acceptable results. Cochran (repeatability) and Grubbs (reproducibility) outlier tests revealed 16 outlier results out of a total of 260 results, as follows: Laboratory 3 had two Grubbs outlier results, five Cochran’s test outlier results, and two results were not reported (negative values provided). Laboratory 7 had two Grubbs outlier results, three Cochran’s test outlier results, and one result was not reported (negative value provided). A single Cochran’s test outlier result was reported outside of Laboratories 3 and 7, by Laboratory 8. Data with calculated means and precision values are shown in Table 3. The average lactose contents ranged from 2.267 mg/100 mL (0.002% lactose) for 6VVU/273B to 235.36 mg/100 mL (0.235% lactose) for 575E/Q2CA. All materials had repeatability relative standard deviations (RSD_r_) <7%. The reproducibility relative standard deviation (RSD_R_) varied from 3.801% for DL4A/EAN7 to 14.898% for JAV2/6C6R, with 7 of the 10 test samples having an RSD_R_ of <10%.

**Table 1.** Interlaboratory study results for lactose in low-lactose and lactose-free milk, milk products, and products containing dairy ingredients (LOLAC Method)

Lactose, mg/100 mL										
Sample	Lactose-free milk		Lactose-free milk		Lactose-free milk		Lactose-free milk		Low lactose milk	
	6VVU & 273B		JAV2 & 6C6R		C886 & BBJ3		B24Z & QVHG		DL4A & EAN7	
Lab no.										
1	2.546	2.888	5.623	5.855	6.503	6.394	14.490	13.609	159.327	163.022
2	2.283	2.280	5.746	5.768	6.626	6.610	13.644	13.526	166.595	163.112
3	2.153	1.855	4.430	1.331^a^	3.761^a^	5.725	14.315	3.724^a^	157.843	155.224
4	2.233	1.868	4.757	4.466	6.474	6.321	12.780	13.689	155.341	155.632
5	2.509	2.597	5.841	5.557	6.721	6.794	13.348	13.580	158.454	160.171
6	1.868	2.095	3.807	4.329	6.248	6.304	13.660	13.656	160.532	155.632
7	1.637	1.833	5.026	4.743	5.565^a^	6.830	12.366	12.256	156.767	144.837
8	2.480	2.495	6.466	6.234	7.048	7.150	14.620	14.548	174.573	169.656
9	2.771	2.779	4.233	4.379	6.052	5.994	14.075	14.330	160.520	159.734
10	2.073	2.102	4.655	4.677	6.037	6.132	13.217	13.071	163.138	164.302
11	1.653	1.533	5.540	5.256	6.341	6.039	14.439	13.026	154.480	155.335
12	1.942	2.175	3.957	4.648	6.459	6.226	14.140	13.908	170.325	174.718
13	2.060	2.061	4.702	4.872	6.186	6.208	12.706	13.011	157.392	159.880

a Cochran outlier.

**Table 2.** Interlaboratory study results for lactose in low-lactose and lactose-free milk, milk products, and products containing dairy ingredients (LOLAC Method)

Lactose, mg/100 mL										
Sample	Low-lactose milk		Lactose-free milkshake		Lactose-free infant formula		Lactose-free infant formula		Lactose-free nutritional drink	
	575E & Q2CA		T3RC & 3VXP		YJ2E & WYB8		2NUB & 4JDP		ZTB2 & N2Z6	
Lab no.										
1	240.532	252.724	3.164	3.128	13.189	12.220	18.233	18.517	19.153	20.216
2	231.641	236.276	2.908	3.109	12.649	13.043	16.869	17.139	18.360	19.186
3	245.653	215.336	2.328	1.448^b^	6.105^a^	5.672^a^	NR^c^	NR	12.502^b^	16.917
4	219.322	208.586	2.633	2.640	11.784	11.687	17.751	19.430	18.811	18.560
5	240.765	236.954	2.953	2.851	12.873	13.590	15.352	16.070	18.206	18.577
6	210.969	221.009	3.254	3.299	13.947	12.554	19.886	20.637	19.805	18.838
7	208.818	233.986	2.670	2.859	1.741^b^	NR	9.854^a^	1.203^a^	18.909	6.916^b^
8	253.160	261.248	3.426	3.048	13.057	11.651	18.637	29.770^b^	18.353	19.105
9	241.260	238.787	2.728	2.699	14.034	12.740	16.779	16.630	18.539	19.045
10	236.168	234.422	2.408	2.619	12.084	11.703	15.877	16.482	17.863	18.260
11	231.830	233.125	2.797	2.521	12.105	15.034	17.464	17.935	20.799	19.829
12	249.232	251.676	2.779	2.793	12.220	12.123	18.379	17.603	18.961	19.252
13	222.144	226.089	2.494	2.581	11.540	10.558	15.987	16.276	16.797	16.853

a Grubbs outlier.

b Cochran outlier.

c NR = Not reported.

**Table 3.** Interlaboratory study results for lactose in low-lactose and lactose-free milk, milk products, and products containing dairy ingredients (LOLAC Method). in which data from laboratories 3 and 7 were excluded; statistical evaluation according to AOAC INTERNATIONAL statistics format

	Lactose-free milk	Lactose-free milk	Lactose-free milk	Lactose-free milk	Low-lactose milk	Low-lactose milk	Lactose free milkshake	Lactose free infant formula	Lactose free infant formula	Lactose free nutritional drink
Sample	6VVU/273B	JAV2/6C6R	C886/BBJ3	B24Z/QVHG	DL4A/EAN7	575E/Q2CA	T3RC/3VXP	YJ2E/WYB8	2NUB/4JDP	ZTB2/N2Z6
No. of laboratories	11	11	11	11	11	11	11	11	11	11
No. of replicates	22	22	22	22	22	22	22	22	21	22
Mean, mg/100 mL	2.267	5.076	6.403	13.716	161.903	235.360	2.856	12.563	17.520	18.789
s_r_, mg/100 mL	0.129	0.246	0.098	0.427	2.187	4.760	0.122	0.877	0.517	0.475
s_R_, mg/100 mL	0.327	0.761	0.326	0.627	6.154	13.962	0.289	1.009	1.428	0.959
RSD_r_, %	5.700	4.845	1.533	3.115	1.351	2.023	4.264	6.979	2.942	2.527
RSD_R_, %	14.438	14.989	5.086	4.571	3.801	5.932	10.127	8.031	8.124	5.104
HORRAT value	1.444	1.692	0.595	0.599	0.723	1.193	1.048	1.039	1.106	0.702

### Collaborators’ Comments

Helpful comments were made by several collaborators and have been incorporated into the present procedure. For example, during the pre-trial sample analysis, several collaborators requested clarification regarding the volumes used in the *Enzymatic Determination* step. As a result, the procedure was rewritten to clarify that the volume of deionized water in this step should be adjusted depending on the volume of sample used.

During the pre-trial sample analysis, a collaborator failed to record the absorbance values A_4_ and A_5_. After discussion, it became clear that the instruction provided was not sufficient, and the method was rewritten to specify in greater detail how and when to take absorbance measurements.

The collaborator who requested the inclusion of a specific sample type (*see Sample Selection* section) contacted the study director and the chair of the expert review panel (ERP) when submitting their LOLAC analysis data with additional data that had been generated using their own HPAEC-PAD analysis. It is worth noting before further discussion that while the HPAEC-PAD method used is a valuable tool for comparison, it has not been subject to peer-reviewed validation or AOAC process and is not an officially accredited method. Nonetheless, these supplementary data as presented by the collaborator are addressed in the *Discussion* section.

## Discussion

The comparison of HPAEC-PAD and K-LOLAC methods as provided by the collaborator indicated perfect alignment between both methods for low-lactose samples (>100 mg/100 g), but some deviations were apparent when samples near the lactose-free range (<10 mg/100 mL) were tested, confirming the results presented following the single-laboratory validation (SLV) study for the LOLAC method. Extensive research was carried out during the course of the SLV, and the selectivity of the MZ104 enzyme was discussed in that report. In summary, the hydrolysis profiles for a wide range of oligosaccharides commonly found in lactose-free samples were examined, and a number of potentially interfering oligosaccharides [e.g., 1,3-β-D-galactosyl glucose (3-Lac) and 1,3-β-D-galactosyl lactose (3-Gal-Lac)] were reported. These oligosaccharides are hydrolyzed by MZ104 β-galactosidase in the test, and as they do not cause a “creeping” reaction, they are not removed by the Creep Calculator and can therefore give rise to overestimation of lactose content. In the numerous lactose-free samples that were studied as part of the single-laboratory validation, these oligosaccharides were present in relatively minute quantities. These selectivity data were presented in the SLV to the ERP, and the method achieved First Action status despite these minor limitations. The HPAEC-PAD data presented by the collaborator support the findings discussed in the SLV.

To put the overestimation into context—the sample that was specifically requested by the collaborator (6VVU/273B) returned a value of 0.9 mg/100 mL in their HPAEC-PAD method, and the value the collaborator returned as part of the K-LOLAC MLV for this sample was 2.1 mg/100 mL. The authors also sent this sample to a reputable, ISO-accredited commercial laboratory for contract analysis using their internal HPAEC-PAD/enzyme treatment method for the measurement of lactose in lactose-free products. This sample was reported as containing <5 mg/100 mL. Given that the lactose content in this particular sample lies well below the lower limit specified by the SMPR (10 mg/100 mL), in the view of the method authors, all three methods of analysis can be considered to be in broad agreement.

Despite the potential for overestimation in samples that contain certain oligosaccharides (most notably, 3-Lac and 3-Gal-Lac), this test remains the most selective enzymatic/spectrophotometric test for lactose in lactose-free products. There was agreement within the ERP discussions that it would be beneficial to bring an enzymatic method through the approval process to cater to the many laboratories that do not have access to HPAEC-PAD or other costly instrumental techniques, and the LOLAC assay delivers on this objective.

All materials had repeatability relative standard deviations (RSD_r_) <7%. The reproducibility relative standard deviation (RSD_R_) varied from 3.8 to 14.9% with 7 of the 10 test samples having an RSD_R_ of <10%. This method meets the requirements for reproducibility set out under SMPR 2018.009.

### Recommendation

On the basis of the results of this study, it is recommended that the spectrophotometric method for lactose concentration in low-lactose and lactose-free milk, milk products, and products containing dairy ingredients be adopted as a Final Action *OfficialMethod*^SM^. Some overestimation of lactose concentration may occur in samples containing 3-Lac and 3-Gal-Lac.
